# Postharvest biocontrol agents in fruits and vegetables: mechanisms, applications, challenges, and future perspectives

**DOI:** 10.3389/fnut.2026.1929304

**Published:** 2026-08-20

**Authors:** Esa Abiso Godana, Gerefa Sefu Edo, Sebahat Oztekin, Qiya Yang, Kaili Wang, Hongyin Zhang

**Affiliations:** 1 School of Food Science and Engineering, Jiangsu University, Zhenjiang, China; 2 Department of Food Engineering, Faculty of Agriculture, Atatürk University, Erzurum, Türkiye

**Keywords:** biocontrol, disease management, essential oil, fruit and vegetable, microbial antagonist, natural compound

## Abstract

Postharvest losses of horticultural produce, primarily due to microbial decay, remain a major challenge to global food security, accounting for 35–50% of production annually. To control this, overuse of synthetic fungicides has led to pathogen resistance, environmental contamination, and health concerns, prompting a shift toward sustainable biocontrol agents (BCAs). This review comprehensively examines the potential of microbial antagonists (yeasts, bacteria, and fungi), plant-based agents (essential oils and extracts), and natural compounds (e.g., chitosan, alginate, organic acids, etc.) for managing postharvest diseases in fruits and vegetables. Key mechanisms of action including competition for nutrients and space, production of antifungal metabolites and enzymes, biofilm formation, induction of host resistance, and volatile organic compounds are discussed in detail. Application strategies (pre- and postharvest), synergistic integrations with physical/chemical treatments, advantages over conventional pesticides, and major challenges (e.g., formulation stability, regulatory hurdles, and commercialization) are critically analyzed. Emerging approaches such as omics technologies, microbial consortia, genetic engineering, and nanotechnology offer promising avenues to enhance BCA efficacy and consistency. This review highlights successful examples and future perspectives of BCAs in postharvest diseases control setup and more importantly their co-applications together with other natural disease control methods and technologies. Finally, it underscores BCAs as viable, eco-friendly alternatives that can extend shelf life, preserve quality, and support sustainable postharvest management.

## 1 Introduction

The global demand for fresh horticultural produce is increasing with time due to population growth, health concern, and their nutritional value. However, every year 35–50% of horticultural produce lost due to microbial decay, physiological deterioration, and improper handling. Developing regions such as Africa, Latin America, and parts of Asia have the highest postharvest losses range from 20 to 50%. In Sub-Saharan Africa, for instance, losses for delicate produce such as mangoes, bananas, and tomatoes estimated between 35 and 50%. In certain West African regions, vegetable losses can even surge to 70–80% during peak seasons. However developed regions such as North America and Europe have less postharvest losses relatively estimated between 5 to 20% (1, 2). Reliance only on chemical pesticides raises concerns over their resistance, environmental toxicity, and health risks. Therefore, shift toward nature-based solutions particularly postharvest biocontrol agents (BCAs), has emerged as a transformative strategy to preserve quality, extend shelf life, and align with global food security and sustainability goals (3).

**Figure 1:**
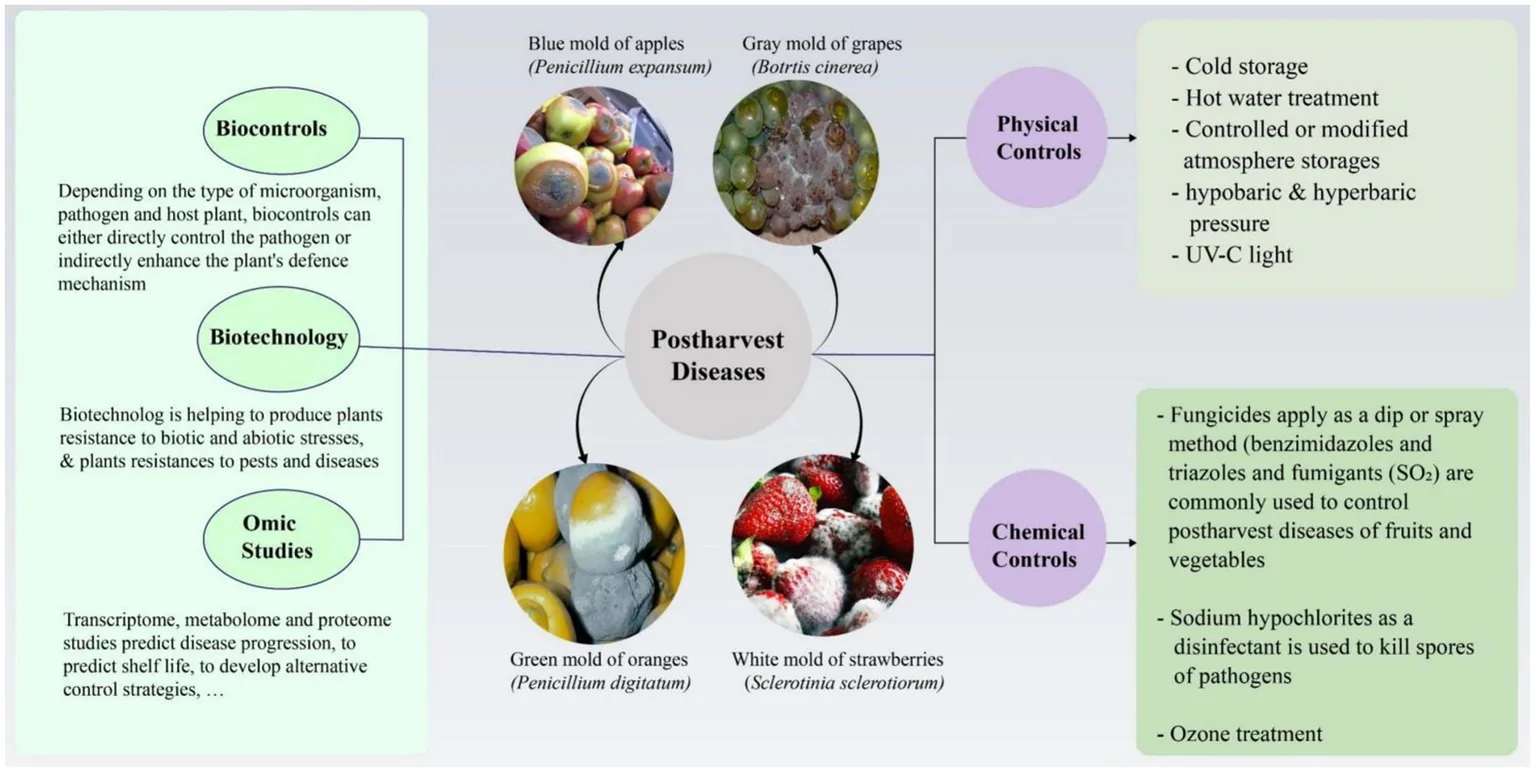
Major postharvest disease control strategies.

Postharvest decay predominantly causes by fungal and bacterial pathogens which thrive in fresh produces nutrient-rich and high-moisture environment (4). To control these decay different methods have been developed. Chemical control remains the primary use followed by physical control such as cold storage strategies. The share of biocontrol and biotechnology is also recently advancing especially in disease control of horticultural produces (5, 6). Figure 1 summarizes some of the major postharvest disease control strategies.

Pathogens such as *Penicillium* spp., *Botryitis cinerea*, *Aspergillus* spp., and *Venturia inaequalis* also showed resistance to fungicides overtime, emphasizing the need for alternative strategies (7–9). Microbial antagonists, their derivatives, and plant-derived compounds suppress pathogen growth and enhance the innate defence mechanisms of fruits and vegetables.

Microbial antagonists engage in competition for nutrients, secrete antifungal metabolites such as lipopeptides and volatile organic compounds, and induce systemic resistance within host tissues. Similarly, plant-derived essential oils, including those from thyme, cinnamon, and oregano, demonstrate broad-spectrum antimicrobial activity through mechanisms such as membrane disruption and inhibition of spore germination. Natural compounds such as chitosan and alginate demonstrate broad disease control efficiency and can preserve postharvest quality of horticultural produces. The integration of biocontrol with complementary technologies has introduced new opportunities in the field. Omics technologies are revolutionizing the discipline by enabling the design of tailored microbial consortia and identifying key pathways involved in pathogen suppression (10).

While laboratory-scale results for biocontrol agents are promising, their commercial-scale application presents significant challenges. The processes of large-scale yeast fermentation, ensuring formulation stability, and achieving regulatory harmonization, particularly concerning genetically modified organisms (GMOs), necessitate interdisciplinary collaboration. Consumer acceptance of novel biocontrol agents is contingent upon transparent communication. The integration of synthetic biology, nanotechnology, and microbiome engineering holds the potential to enhance the precision of biocontrol methods, facilitating dynamic responses to pathogen biomarkers and environmental conditions.

This review article explores promising BCAs identified for managing postharvest diseases in horticultural crops, with a particular focus on fruits and vegetables. It provides a detailed discussion of their mechanisms of action, application methods, advantages, and the primary challenges associated with their practical implementation. Additionally, the review explores their synergistic applications with other postharvest treatments and the future perspectives of the BCAs.

## 2 Major BCAs in postharvest disease management of perishables

Biocontrol agents encompass a diverse array of biological and naturally derived substances used to suppress postharvest pathogens while minimizing environmental and health risks associated with synthetic fungicides (11). Although the term “biocontrol agent” traditionally emphasizes living microorganisms, many reviews adopt a broader definition in postharvest contexts to include plant-derived extracts and natural compounds due to their shared antimicrobial and quality-preserving properties.

In this review, we considered the broader definitions of the BCAs and classified them into three main categories for clarity and comprehensiveness: (1) microbial agents (living antagonistic microorganisms), (2) plant-based agents (essential oils and plant extracts), and (3) natural compounds (e.g., chitosan, alginate, and organic acids). This inclusive approach reflects common practice in postharvest literature, where efficacy against decay and compatibility with sustainable practices often take precedence over strict etymological definitions (12).

### 2.1 Microbial agents

#### 2.1.1 Yeast-based BCAs

Antagonistic yeasts are among the widely studied microbial agents. They have good adaptation ability, most of them are non-toxic, and they have good ability to easily colonize the surface of fruits and vegetables. *Wickerhamomyces anomalus*, *Cryptococcus podzolicus*, *Meyerozyma guilliermondii*, *Cryptococcus laurentii*, *Meyerozyma caribbica*, *Pichia membranefaciens*, etc., are among the few notable antagonistic yeasts that are reported for their potential to control major fungal pathogens, and some of them even degrade mycotoxins produced by them (13).

Different yeasts have different control mechanisms. For example, the antagonistic yeasts *M. guilliermondii* and *C. laurentii* are known for their direct antagonism by secreting antifungal compounds that inhibit the growth of pathogenic microbes (14). Other antagonists such as *P. membranefaciens* are known for their rapid colonization of the fruit surface so that they can compete for nutrient and space with the pathogen, one of the major disease control mechanisms (15). Other yeast-based BCAs are reported for their ability to increase the disease resistance of the host by increasing the accumulation of antifungal compounds and disease defence-related enzymes and are summarized in Table 1.

**Table 1:** Yeast based biological control agents against fruit and vegetable postharvest diseases.

Yeast strains	Target pathogens	Fruits/vegetables	Key references
*Wickerhamomyces anomalus* (e.g., strains Y16, KTCf20)	Grey mold (*B. cinerea*), black spot decay, blue/green mold (*P. expansum*, *P. italicum*), *Alternaria* spp.	Tomatoes, grapes, apples, strawberries, citrus	(24, 46, 47, 51, 64, 65)
*Pichia membranefaciens*	*Rhizopus* rot, *Penicillium* spp., *B. cinerea*	Nectarines, citrus, apples, peaches, tomatoes	(14, 15)
*Meyerozyma guilliermondii* / *Meyerozyma caribbica*	*Penicillium* spp., *Alternaria* spp., *Botrytis* spp., *Colletotrichum* spp.	Citrus, mangoes, grapes, tomatoes	(14, 66)
*Cryptococcus laurentii* / *Cryptococcus podzolicus*	*Penicillium* spp., *B. cinerea*, *Monilinia* spp., *Alternaria* spp.	Apples, pears, strawberries, cherries, citrus	(67)
*Aureobasidium pullulans* (various strains)	*Penicillium* spp., *B. cinerea*, *Colletotrichum* spp., *Monilinia* spp.	Apples, grapes, kiwifruit, strawberries, pears	(67, 68)
*Debaryomyces hansenii*	*Penicillium* spp., *Botrytis* spp., *Rhizopus* spp., soft rot, *Monilinia* spp.	Strawberries, muskmelon, citrus, apples	(14, 69)
*Papiliotrema terrestris* (e.g., PT22AV/YSY®)	*P. expansum*, *B. cinerea*, *R. stolonifer*, *A. niger*	Apples, pears, strawberries, kiwifruit, table grapes	(70, 71)
*Candida oleophila* (commercial, e.g., Aspire)	*Penicillium* spp., *B. cinerea*, *Rhizopus* spp.	Citrus, pome fruits (apples, pears)	(16, 72)
*Pichia caribbica*	Black spot decay (*A. alternata*), *Penicillium* spp.	Cherry tomatoes, citrus	(73, 74)
Hannaella sinensis	*B. cinerea*, *Penicillium* spp.	Apples, strawberries	(75, 76)
*Sporidiobolus pararoseus* (e.g., Y16)	Brown rot (*M. fructicola*), *Penicillium* spp.	Peaches, apples	(50, 77)

#### 2.1.2 Bacterial-based BCAs

Bacterial based BCAs are a real and actively studied option for postharvest disease control of fruits and vegetables. However, most of the evidence are handful of strain-specific *in vitro/in vivo* studies rather than broad commercial validation. The strongest and promising genera are *Bacillus*, *Pseudomonas*, and *Pantoea*, with the clearest targets being fungal postharvest pathogens on citrus, apples, pears, strawberries, and grapes (16–18).

The control mechanisms of these BCAs are consistent and similar in most studies: competition for space and nutrients, antibiosis, siderophores, lipopeptides and other diffusible metabolites, biofilm formation, hydrolytic enzymes, and induced resistance are the main modes invoked. *Pantoea agglomerans* CPA-2, one of promising bacterial BCA gave excellent control of *Botrytis cinerea* and *Penicillium expansum* under cold storage, in some cases matching imazalil under the reported conditions (19). A later apple/pear study found *Pseudomonas syringae* CPA-5 similarly effective in semi-commercial trials (20). More recent work in strawberries and grapes found *Bacillus* spp. and *Pseudomonas fluorescens* UM270 reduced disease incidence and improved firmness, while citrus studies found epiphytic *Bacillus* and *Pseudomonas* isolates active against *Penicillium digitatum* and *P. italicum* (21, 22).

Even though there are lots of candidates that are reported as potential bacterial BCAs what is lacking is the translation. Several reviews explicitly note that widespread commercial use has not materialized, and that strain specificity, formulation, environmental stability, and regulatory/commercial hurdles remain unresolved (23, 24). So the literature supports bacterial BCAs as promising and often effective under controlled storage/experimental conditions, but not yet as a universally dependable plug-in replacement for fungicides.

#### 2.1.3 Fungal-based BCAs

Certain fungal microbes can serve as effective control agents, as they pose no harm to human or animal health. *Trichoderma* species are among the major fungal BCAs and are used as biofungicides to control different postharvest diseases of fruits and vegetables. Nujthet et al. (25) reported that *Trichoderma viride* can control *Alternaria* and *Botrytis* rot during the postharvest stage. *Trichoderma harzianum* was reported for its efficiency in controlling *Aspergillus* and *Fusarium* rots in citrus fruits (26).

Their core control methods are similar to other microbial BCAs and is similar across different strains. Competition for nutrients and space, mycoparasitism/direct parasitism, antifungal antibiotics and volatile metabolites, and induction of host resistance are among the major control mechanisms. Several studies also reported that postharvest application of the BCAs works better than preharvest application, and that their efficacy improves when BCAs are combined with other postharvest disease control methods such as coatings, low-dose fungicides, salt additives, hot water, irradiation, encapsulation, or other enhancement strategies (27, 28).

Generally the idea of using safe fungal BCAs to control postharvest diseases of fruits and vegetables is not at the “does this work at all?” stage; it is at the “which strain, which fruit, which formulation, and which add-on treatment makes it reliable enough to commercialize?” stage. Therefore, future studies should focus on these areas for practical applications (29).

### 2.2 Plant based agents

Plant-based agents such as essential oils and plant extracts, have emerged as eco-friendly alternatives to synthetic fungicides for managing postharvest diseases of fruits and vegetables. They have bioactive compounds that have antimicrobial, antioxidant, and elicitor properties to control the pathogen growth and preserve quality of perishable crops (30).

#### 2.2.1 Essential oils

Essential oils (EO) are mostly extracted from aromatic plants. They are rich in volatile phenolic compounds, terpenoids, and aldehydes, which exhibit broad-spectrum antimicrobial activity. For example, thyme EO contains thymol and carvacrol, which can grow postharvest grey mold and green mold in strawberries and citrus, respectively (31). Oregano EO, which also has carvacrol, exhibited strong antifungal properties against *Rhizopus stolonifera* and *Aspergillus flavus* in peaches and tomatoes. EO extracted from clove (eugenol) can also inhibit the fungal growth of *Colletotrichum gloeosporioides* in mangoes and avocados (32, 33).

#### 2.2.2 Plant extracts

Extracts from some plant leaves, seeds, roots, and peels contain polyphenols, flavonoids, and alkaloids that have antioxidant and antimicrobial functions. For example, extracts from neem leaves contain nimbin and azadirachtin, which inhibit the fungal growth and spore formation of *A. alternata* and *F. oxysporum*. Peel extract from pomegranate also exhibited high content of ellagic acid and punicalagins that suppress the growth of *B. cinerea* (34). Plant extracts from green tea, *aloe vera*, and numerous other plants also demonstrated strong antifungal properties and can be used as an alternative postharvest treatment if formulated (35).

### 2.3 Natural compounds

Consumer interest toward organic consumption is increasing occasionally, and the demand for using natural compounds as postharvest treatment is increasing. Numerous studies report the potential of biopolymers such as chitosan and alginate to control fruits and vegetables postharvest pathogens (36). Crustacean shells and fungal cell walls can yield chitosan, a linear polysaccharide. Its antimicrobial characteristics arise from its polycationic nature and can inhibit the spore germination of pathogenic microorganisms (37, 38). Romanazzi et al. (39), reported that chitosan can inhibit the growth of *B. cinerea* by up to 80% in strawberries.

A seaweed-derived biopolymer alginate has the ability to form gel and is widely used in edible coatings. Combining alginate with essential oils increases their stability and slows release. The combination of alginate together with lemongrass oil was reported for its high antifungal properties against *P. digitatum* with a control rate of up to 90% (40).

Organic acids such as citric acid and acetic acid are effective antimicrobial agents that lower pH and disrupt microbial enzyme systems. Citric acid, a tricarboxylic acid found in citrus fruits, chelates metal ions essential for pathogen survival. Dipping apples in 2% citric acid solutions reduced *M. fructicola* infections by 70% and delayed browning (41). Acetic acid, a short-chain fatty acid, exhibits broad-spectrum antifungal activity. Vapor-phase acetic acid demonstrated effective postharvest diseases control of fruits and vegetables (42).

## 3 Role of BCAs in postharvest disease management and quality preservation

BCAs play a crucial role in managing postharvest diseases and preserving the quality of fruits and vegetables even if their market share is currently insignificant. These agents, could definitely replace chemical pesticides if their efficiency is equal or near to equal of the chemicals. The following are major roles of the BCAs in postharvest disease management and quality preservation.

### 3.1 Disease control

Numerous BCAs are reported for their efficiency to control a wide range of postharvest pathogens such as *Alternaria*, *Botrytis*, *Colletotrichum*, *Monilinia*, *Penicillium*, and *Rhizopus* spp. (20). Table 2 summarizes the major BCAs and their role in postharvest disease control of different horticultural produces.

**Table 2:** Major BCAs identified to control postharvest diseases of fruits and vegetables.

Types of BCAs			Target pathogens	References
Microbial agents	Yeasts	*Pichia* spp.	*Penicillium* spp., *Alternaria* spp., *Botryitis* spp., etc.	(51, 64, 65)
		*Debaryomyces* spp.	*Penicillium* spp., *Botrytis* spp., *Rhizopus* spp.*, Monilinia* spp., etc.	(14, 69)
		*Aureobasidium* spp.	*Penicillium* spp., *Botrytis* spp., *Colletotrichum* spp., *Monilinia* spp., etc.	(67, 68)
	Bacteria	*Bacillus* spp.	*Penicillium* spp., *Botryitis* spp., *Sclerotinia sclerotiorum*, etc	(78–80)
		*Pseudomonas* spp.	*Xanthomonas axonopodis*	(81)
	Fungi	*Trichoderma* spp.	*Sclerotium cepivorum, Meloidogyne incognita, Colletrotrichum gloeosporiodes,* etc.	(82, 83)
		*Diaporthe apiculatum*	*Alternaria* spp., *Botyritis* spp., *Colletotrichum* spp., etc	(84)
Plant-based agents	Essential oils	Thyme	*Penicillium italicum*	(85, 86)
		Oregano	*B. cinerea*	(86)
		Clove	*P. italicum*	(87)
	Plant extracts	Neem	Numerous fungal pathogens	(88)
		*Aloe vera*	*Penicillium digitatum*	(89–91)
Natural compounds	Biopolymers	Chitosan	Control numerous postharvest diseases of fruits and vegetables	(39, 92, 93)
		Alginate	*Monilinia fructicola* and other major diseases	(77, 94)
	Organic acids	Citric acid	Delay ripening, browning, and control disease development in numerous fruits and vegetables	(42, 95)
		Acetic acid	*B. cinerea*, *P. expansum,* etc.	(42, 96, 97)

### 3.2 Quality preservation

The maximum quality of perishable crops is at the time of harvest and it is important to retain that quality during postharvest stage. As discussed in Section 2.2 and 2.3, essential oils, plant extracts, and natural compounds are usually used to preserve the quality of fruits and vegetables. BCAs can reduce disease incidence and maintain the physicochemical properties, sensory attributes, and nutritional value of fruits and vegetables, thereby extending their shelf life (43).

### 3.3 Environmental benefits

Currently chemical pesticides are the primary approach to control postharvest disease and preserve quality of fruits and vegetables with an estimated increase in chemical use by up to 1500% since 1996 (44). These widely brings the concerns of environmental issues, food safety, and development of disease resistant pathogens. Compared to synthetic fungicides, BCAs are naturally occurring and they are less harmful to humans and the environment making them a sustainable choice for postharvest management (45).

## 4 Mechanisms of action

**Figure 2:**
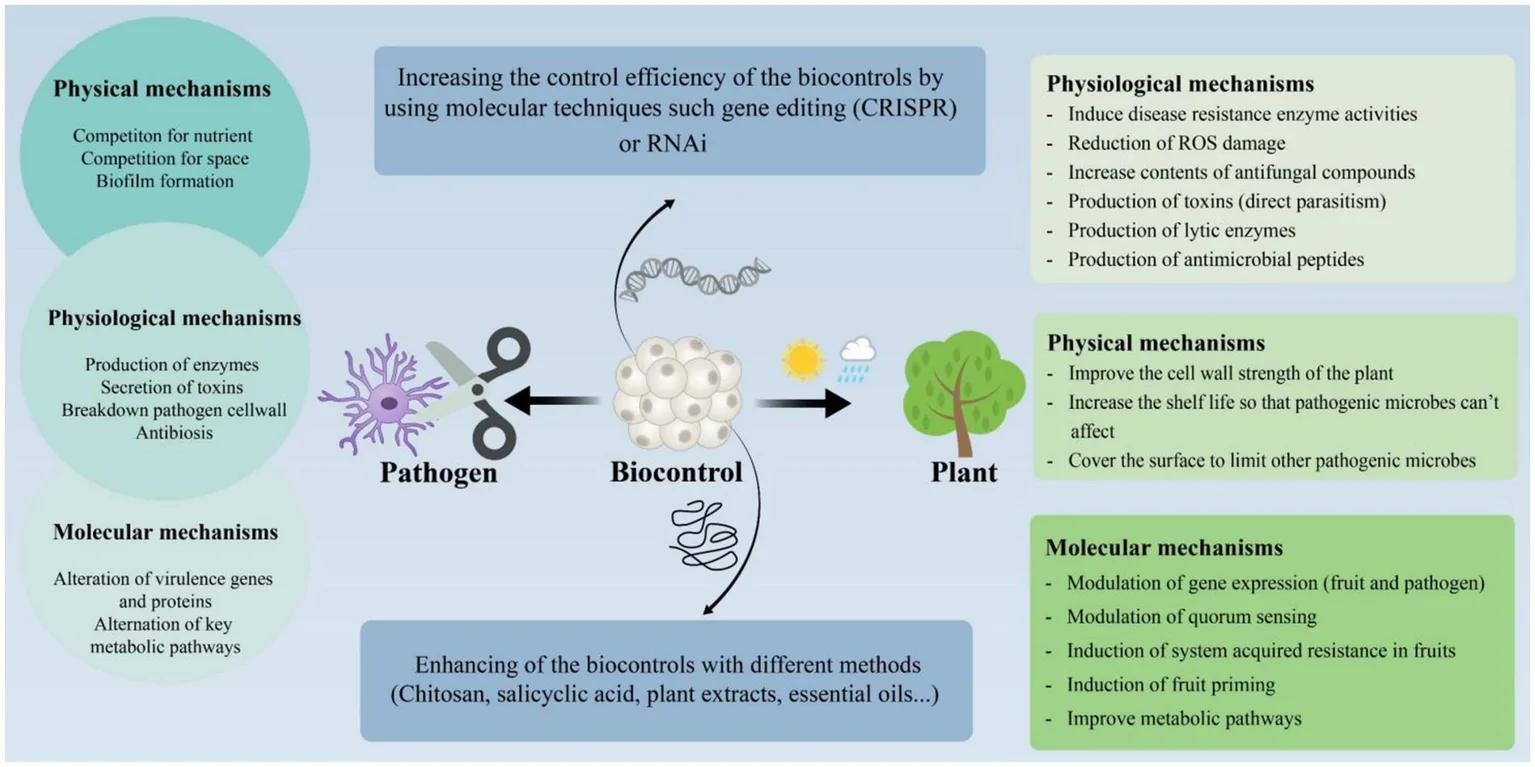
The physical, physiological, and molecular mechanisms by which BCAs control postharvest diseases of fruits and vegetables.

The mechanisms by which BCAs inhibit mold growth may involve direct suppression through the production of antifungal substances and metabolites or by degrading the fungal cell wall. Additionally, BCAs may modify the environment by altering the pH, competing for resources, or physically occupying space. Another potential mechanism is the enhancement of the host disease defence potential. Generally, we can classify these mechanisms into three: Physical mechanisms include actions such as physical barriers (biofilm formation) and competition for nutrients and space; physiological mechanisms include improving the host’s defence ability by increasing the accumulation of antifungal compounds, enzymes, etc.; and the molecular mechanisms include improving the metabolic activities of the host. Figure 2 summarizes the major physical, physiological, and molecular mechanism actions of BCAs both in the host and the pathogen.

### 4.1 Production of volatile compounds

Numerous microbial antagonistic are reported for their production of volatile compounds which can suppress the growth of fungal pathogens. The major volatile organic compounds produced by antagonistic yeasts are ethanol, ethyl acetate, isoamyl alcohol, isoamyl acetate, phenyl acetate, butanoic acid-ethyl ester, ethyl caprylate, 2-phenylethanol, 2-methyl-1-propanol, acetic acid-butyl ester, 3-methyl-butanoic acid, benzyl alcohol, etc. (46). These VOCs can be used as bio-fumigants because they do not directly contact the pathogen or the food. Therefore, VOCs produced by these antagonistic microbes can be more realistically used in the practical setup.

### 4.2 Production of killer toxins

Microbial BCAs can produce toxic compounds such as nitrophenol derivatives, which can suppress the growth of different pathogenic fungi and bacteria. For example, a popular antagonistic yeast *W. anomalus* can produce killer toxin (also known as mycocin) KTCf20 which can control numerous postharvest pathogens and air-borne fungi (47). The production of killer toxins and other metabolites which can suppress the growth of pathogenic microbes are the main characteristics of microbial BCAs (48, 49).

### 4.3 Production of degrading enzymes

Microbial BCAs are known for their production of hydrolytic enzymes which can degrade the cell walls of mold. Chitinases and glucanases are among the major degrading enzymes produced by the antagonistic yeasts.

### 4.4 Biofilm formation

Certain bacteria and yeasts can form biofilms on the surface of produce, creating a protective barrier against pathogen colonization and also increase stress tolerance. These biofilms not only physically block pathogens but also secrete antimicrobial compounds that inhibit their growth. A strain of *W. anomalus* was reported for its high production of biofilm [up to 0.3025 at 600 OD while the control was only 0.0979; (49)].

### 4.5 Induce host disease resistance

Postharvest application of BCAs to horticultural produce can also increase the host disease resistance by improving the accumulation of antifungal compounds such as phenols and flavonoids. Numerous studies reported that, antagonistic yeasts can increase the activities of disease defence related enzymes, increase the accumulation of antifungal compounds and metabolic activities in fruits and vegetables (50, 51).

### 4.6 Competition for nutrients and space

BCAs often outcompete pathogens for essential nutrients and physical space on the surface of fruits and vegetables. This competitive exclusion limits the resources available for pathogenic fungi such as *P. expansum*, *B. cinerea*, and *A. alternata*, thereby reducing their ability to colonize and infect the produce. For example, certain antagonistic yeasts and bacteria have been shown to rapidly colonize fruits and vegetables surfaces, creating a physical barrier that prevents pathogen attachment and growth (52, 53).

## 5 Application methods

**Figure 3:**
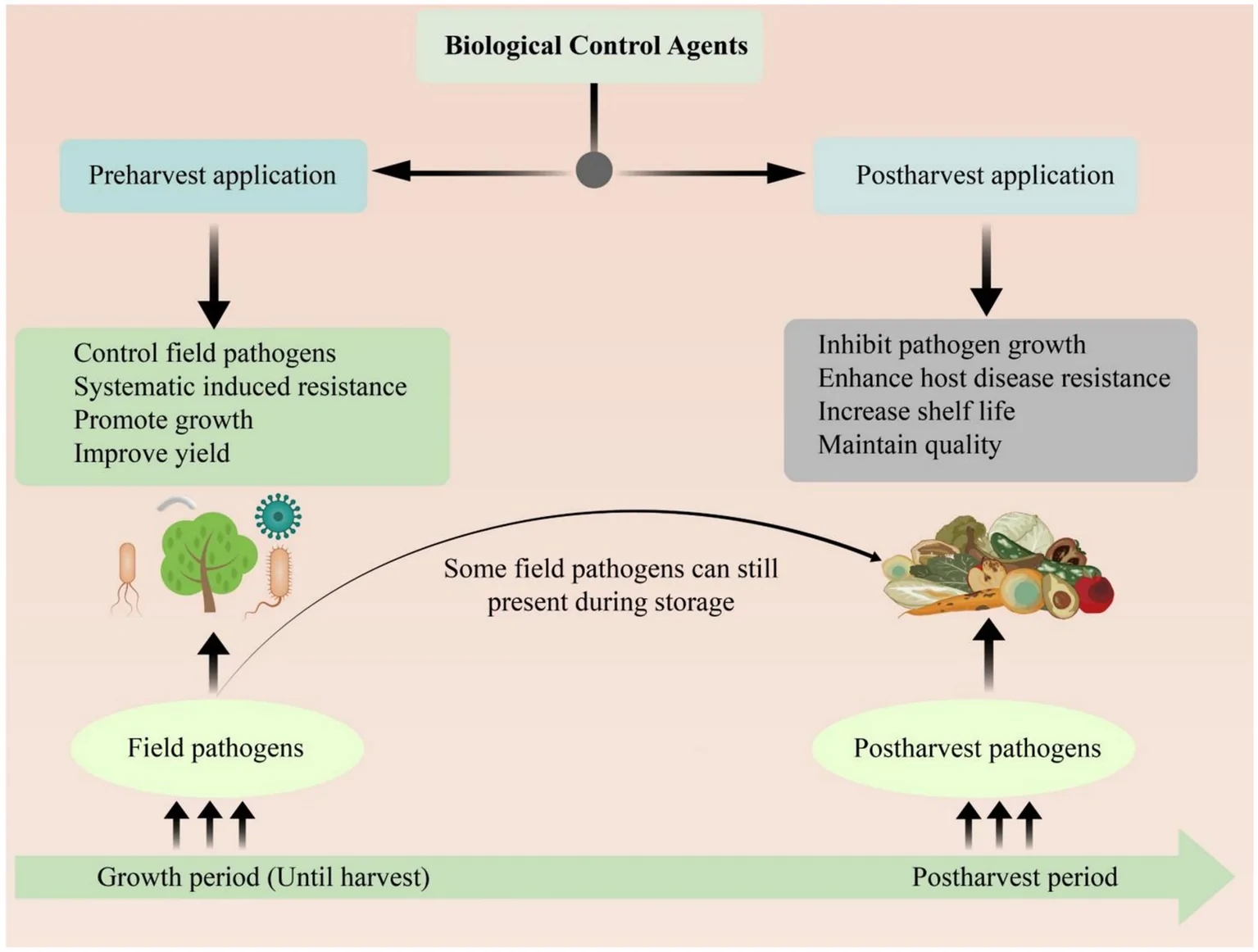
BCAs application strategies during preharvest and postharvest periods.

BCAs can be used during preharvest or postharvest periods. Generally, their application is more efficient during postharvest due to stable environmental condition at storage. They can also be used as a combination approach with other compatible disease control methods. The preharvest and postharvest applications of BCAs are summarized in Figure 3.

### 5.1 Pre-harvest application of BCAs

BCAs can be applied at the field to control or reduce the initial microbial load of fruits and vegetables before they are harvested. Some of the BCAs are also known to boost the plant disease resistance immunity and promote growth. Field application of BCAs are also effective to reduce the risk of postharvest diseases. For example, *A. pullulans* has been used to control *B. cinerea* on grapes and kiwifruit during pre-harvest stages (54). BCAs can be applied directly to crops in the field through spraying or foliar application. Others can be applied to the soil or root area to induce plant immunity and reduce colonization of pathogenic microbes. However, application of BCAs in preharvest setup is less effective compared to the postharvest setup (which is relatively stable environment) due to rainfall leaching, field microbial interference, short duration of efficacy, unstable weather, etc.

### 5.2 Postharvest application of BCAs

Currently numerous BCAs, especially antagonistic yeasts are reported for their efficiency to control postharvest diseases of fruits and vegetables. Different natural compounds such as chitosan, salicylic acid, trehalose, etc. can even improve their efficiency. The combine applications of antagonistic microbes are also exhibiting better result than using solo application.

In addition to disease control, postharvest application of BCAs can retain the quality and extend shelf life of fruits and vegetables. They can be applied through various methods such as dipping, spraying, coating, or incorporation into edible films. Currently studies in the area of BCAs formulation and application are very limited which delays their commercial application.

Dipping and spraying can be applicable in stone and citrus fruits. *B. subtilis* and chitosan have been applied to citrus fruits to control green mold decay (54). BCAs can be incorporated into edible coatings made from biopolymers such as chitosan, alginate, or carnauba wax. These coatings not only provide a physical barrier but also deliver BCAs directly to the produce surface apples (55).

Probiotic bacteria such as *Lactobacillus rhamnosus* and *Bifidobacterium animalis* have showed excellent results when added to alginate-based coatings that are enhanced with oligofructose and inulin prebiotics. These coatings demonstrated bactericidal activity against *Listeria innocua* and inhibited the development of *Escherichia coli* and preserved nutritional quality when applied to fresh-cut apples (56).

### 5.3 Combined applications

The combined application of BCAs with other methods is a promising strategy to control postharvest diseases. Integrating BCAs with physical treatments (e.g., heat, irradiation) or chemical treatments can improve their efficiency against fungal pathogens such as *Penicillium*, *Monilinia*, and *B. cinerea* (57).

This approach overcomes the limitations of BCAs when used alone, such as inconsistent activity and insufficient disease control (52). Edible coatings combined with BCAs offer a synergistic effect on pathogen suppression and quality maintenance (55). Additionally, integrating BCAs with chemical fungicides can reduce fungicide doses and mitigate resistance development in pathogens. However, challenges remain, including optimizing application techniques, ensuring compatibility between treatments, and addressing regulatory requirements.

## 6 Advantages, challenges, and limitations of BCAs

### 6.1 Advantages of BCAs in the control of postharvest diseases

BCAs have emerged as a sustainable and effective alternative for managing postharvest diseases of fruits and vegetables. The key advantages of using BCAs in postharvest disease management over synthetic chemical pesticides are summarized in Table 3.

**Table 3:** Advantages of BCAs over synthetic chemical fungicides in control of postharvest diseases of fruits and vegetables.

Advantages	Description	References
Environmentally friendly	BCAs are natural or are derived from natural sources and are biodegradable. They do not leave harmful residues on produce or in the ecosystem, making them a safer option for sustainable agriculture.	(98)
Safe for human and animal health	Because BCAs are non-toxic to both humans and animals, regulatory bodies have classified them as generally recognized as safe (GRAS).	(99)
Reduced risk of pathogen resistance	Unlike chemical fungicides, BCAs employ multiple mechanisms of action such as competition for nutrients and space, production of antimicrobial compounds, and induction of host resistance. This multifaceted control approach minimizes the likelihood of pathogens to develop resistance.	(100)
Compatibility with IPM and organic farming	BCAs can be integrated with low-toxicity chemicals, heat treatments, and controlled environment storage, among other disease management techniques. In addition, BCAs are compatible with organic and sustainable agriculture systems.	(101)
Induce host disease defence ability	Some BCAs stimulate the host defence ability. For example, by stimulating the production of phytoalexins and pathogenesis-related (PR) proteins.	(72)
Wider range of applications	BCAs have wider range of applications in postharvest disease management of fruits and vegetables. Therefore, they can be better incorporated into organic farming practices.	(102)
Consumer acceptance	Consumers interest toward organic and residue free consumption is increasing from time to time. So, BCAs have higher consumer acceptance than chemical pesticides.	(103)
Commercialization	With the increasing utilization of biotechnology in food and agriculture, the chance of rapid commercialization of BCAs are high. BCAs such as *Candida oleophila* (Aspire) and *Bacillus subtilis* (Serenade), are currently commercially available.	(16)

### 6.2 Challenges and limitations

**Figure 4:**
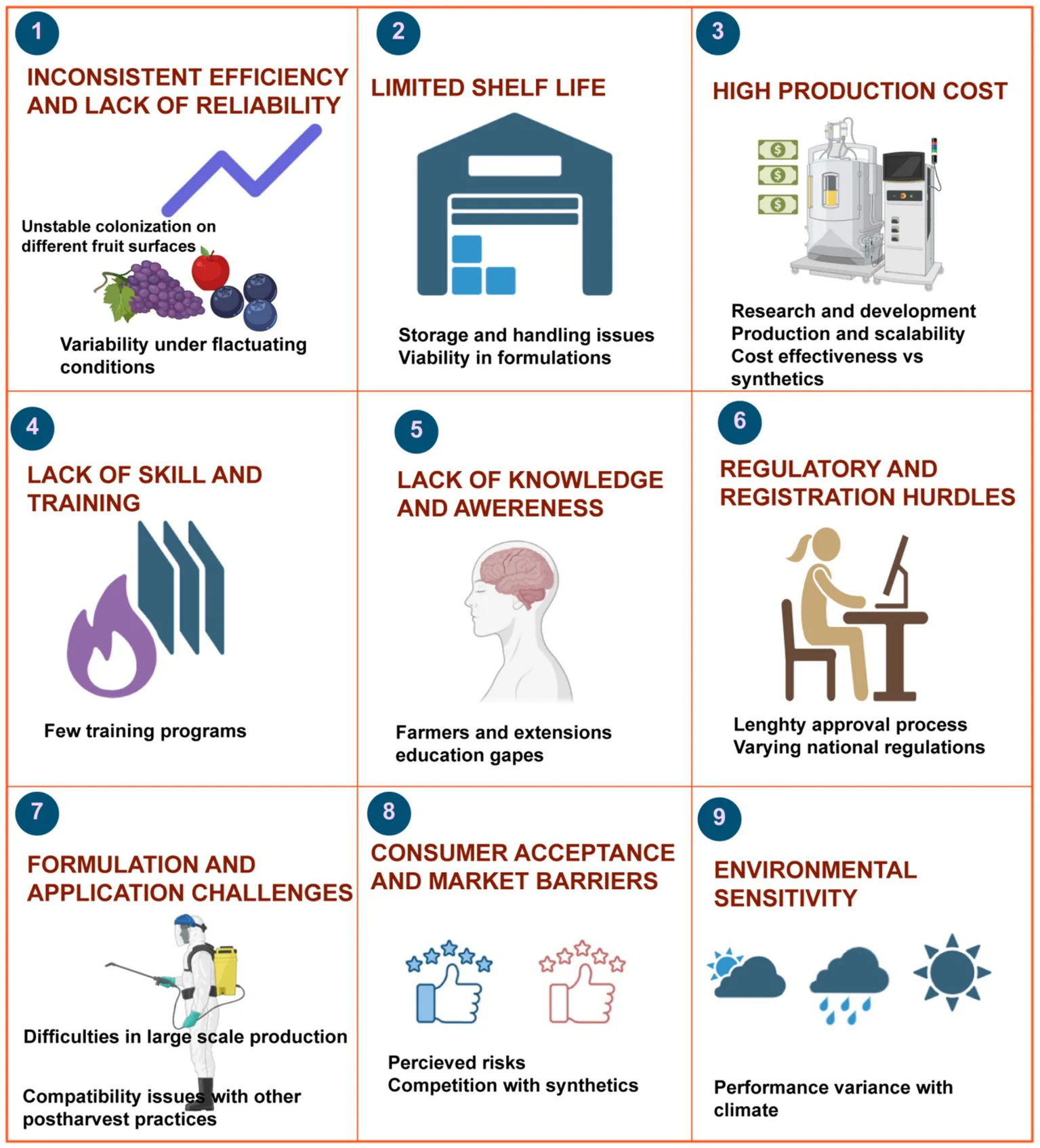
Major challenges and limitations in the use of biological control agents in postharvest applications.

While BCAs have many advantages and demonstrated good potential in controlling postharvest diseases of fruits and vegetables, comparing to the synthetic chemical pesticides the commercial market share is very low. Challenges and limitation such as regulatory, technical, and social factors limit their efficacy, commercialization, and acceptance. Figure 4 summarizes the major challenges and limitations of BCAs in postharvest applications.

## 7 Future perspectives of BCAs

Recent investigations into BCAs have identified several promising directions and challenges that are likely to influence their future practical application. Although BCAs such as BioSave and Aspire have already been commercialized, their continued success depends on addressing variability in field performance, increasing users’ awareness, and fostering collaborations between researchers and industry stakeholders. This section examines the technological integrations and challenges that are expected to shape BCA strategies for horticultural produce over the coming decades.

### 7.1 Integration with emerging technologies

The application of omics technologies is significantly advancing the understanding of biocontrol mechanisms. For example, mechanisms such as induced systemic resistance and the modulation of hormone signaling pathways are among the primary mechanisms identified in various studies. CRISPR-based gene editing holds potential for enhancing antagonistic traits in yeasts, including the production of antifungal compounds, stress tolerance, and biofilm formation. Genetic engineering is being investigated to improve the efficacy of BCAs, such as by enhancing stress tolerance or antifungal compound production in different strains including *Bacillus subtilis* and *Trichoderma* spp. (58). The application of double-stranded RNA (dsRNA) to silence pathogen genes presents a targeted approach that does not adversely affect beneficial microbes or the environment (59). Furthermore, metabolomic profiling of plant-derived antifungals, such as essential oils, could optimize formulations to enhance stability and target specificity.

### 7.2 Systems-based approaches

Future research should prioritize the utilization of microbial consortia over single-strain applications for the control of diverse pathogens. For instance, the microbiome of wild apples (*Malus trilobata*) has revealed novel genera such as *Bosea* and *Rhizobium*, which exhibit antifungal activity against *B. cinerea* and *P. expansum* (60). Similarly, non-*Saccharomyces* yeasts, including *Aureobasidium melanogenum* and *Pichia kluyveri*, have demonstrated the ability to inhibit three different strains of *B. cinerea* on strawberries and apples, indicating that tailored consortia could effectively target multiple pathogens simultaneously (61).

Understanding the interactions between BCAs, pathogens, and the resident produce microflora, referred to as the “pathobiome concept,” is crucial. Tailoring BCAs to synergize with native microbial communities could enhance consistency across varying environmental conditions (59). The integration of BCAs with other postharvest methods, such as physical treatments, low-dose fungicides, or natural compounds, could enhance control efficacy and reduce reliance on synthetic chemical pesticides (62). Numerous recent studies aimed at improving the control efficiency of BCAs through the use of natural compounds such as trehalose, chitosan, phytic acid, etc. have shown promising developments (24, 50).

### 7.3 Commercialization and practical challenges

Despite promising results at the laboratory scale, the commercialization and practical application of BCAs remain challenging, with a market share of less than 1%. The primary obstacles include optimizing large-scale yeast fermentation, ensuring shelf-stable formulations, and navigating regulatory frameworks. Although certain BCAs have demonstrated postharvest disease control efficacy exceeding 50%, their commercial adoption necessitates validation under diverse storage conditions.

Harmonizing global regulations for biocontrol products, particularly those derived from genetically modified organisms (GMOs), is critical for market entry. Collaborative efforts among academia, industry, and policymakers can expedite this process. Developing stable, shelf-ready products through nanoencapsulation or improved carrier materials remains a priority to enhance BCAs’ viability during storage and application. Streamlining approval processes and demonstrating cost-effectiveness compared to conventional methods are essential for scaling up the practical use of BCAs (52, 63).

## 8 Conclusion

The postharvest application of BCAs generally reduces decay and extends shelf life, although their effectiveness varies depending on the commodity and pathogen. The pre-harvest use of BCAs is less common due to environmental variability. Several studies have demonstrated that integrating microbial antagonists with low-risk chemical fungicides, natural plant products, or physical treatments (e.g., hot water dips or UV irradiation) enhances disease control outcomes. Furthermore, emerging approaches such as genetic modification, microbiome-based strategies, RNA-based treatments, and enhanced formulations offer full potential uses of the BCAs. Future researches should focus on combined applications of BCAs with these technologies and methods. Furthermore, improving the efficacy of BCAs and large-scale production or commercial application needs extensive studies beyond the lab trials. Extending the shelf life and retaining postharvest quality of fruits and vegetables are important factors to maintain their nutritional value.
